# Human Effort in Polar Climates

**Published:** 1913-02-22

**Authors:** 


### HUMAN EFFORT IN POLAR CLIMATES.

When the full story of Captain Scott's ill-fated journey is published the exact causes of the disaster may be made plain, and in the meanwhile speculation concerning them is idle. But there are well-known facts established by the observations of past generations of explorers in polar climates which are applicable to any incursion by human beings into the regions of perpetual cold.

Below the freezing-point there is an excessive demand for the production of heat within the body, because the difference in temperature between the blood and surrounding objects is greater. This demand naturally increases with every fall of external temperature below the freezing-point, and it has to be met by an increase in the total heat-producing capacity of the food taken. Thus both the total quantity and the quality of the food which an Arctic explorer needs is greater than that of the same man in a temperate clime. In practice it is difficult to supply the whole of this extra heat-producing food, and the result is that explorers have to live on themselves to a great extent—that is, to get very thin. This effect of the low temperatures at which they live is accentuated by the strong winds which they may encounter, for these enormously increase the heat-abstracting power of a low temperature. Another factor which adds yet further to the result of the first two is the very severe physical work demanded by travel and sledge-hauling on glaciers or over obstructions of any sort.

It is easy to see that endurance and vitality of no ordinary kind are demanded of a polar traveller. Reduced to the lowest ebb of strength by their long period of overwork and starvation, Scott's party were unable to get that fatal eleven miles which alone separated them from an ample depôt of food and stores; and the protracted blizzard which they encountered completed their ruin. Heroism such as theirs is not easy on a full stomach and before a gallery; but in their condition and situation it becomes one of the most sublime acts in the story of our race. Captain Oates's deliberately going out to certain death to give his leader and the party a better chance of winning through is a deed which deserves, and will, we trust, obtain, the immortality of deathless verse. Mr. Kipling is, perhaps, the man to do it justice, and we hope he will try. Magnificent as such self-sacrifice would be in any circumstances, it transcends our imagination when accomplished by a famished, worn-out man on the verge of physical collapse. But the whole story of this gallant company is equally inspiring, and is a testimony to the effects of Antarctic environment than which nothing more impressive and nothing more catastrophic could well be conceived.

